# Antimicrobial susceptibility testing in 90 min by bacterial cell count monitoring

**DOI:** 10.1111/j.1469-0691.2012.03800.x

**Published:** 2012-02-09

**Authors:** M A C Broeren, Y Maas, E Retera, N L A Arents

**Affiliations:** 1 Máxima Medical Centre, Clinical Laboratory for Chemistry and HaematologyDL Veldhoven, the Netherlands; 2 Department of Medical Microbiology, Laboratory for Pathology and Medical Microbiology (PAMM)DL Veldhoven, the Netherlands

**Keywords:** Antibiotics, cell count monitoring, flow-cytometry, microbiology, susceptibility testing

## Abstract

The rise in antimicrobial resistance has become a serious global health problem. Restrictive use of antibiotics seems the only option to temper this accession since research in new antibiotics has halted. Antimicrobial stewardship programmes rely on quick access to susceptibility data. This study evaluated the concept of bacterial cell count monitoring as a fast method to determine susceptibility. *Escherichia coli*, *Pseudomonas aeruginosa* and *Staphylococcus aureus* strains were tested for amoxicillin/piperacillin and gentamicin by three conventional methods (VITEK2®, Etest® and broth-macrodilution). Bacterial cell count monitoring reliably predicted susceptibility after 90 min for *Escherichia coli* and after 120 min for *Pseudomonas aeruginosa* and *Staphylococcus aureus* without any minor, major or very major discrepancies. Time-to-result was reduced by 74%, 83% and 76%, respectively. Bacterial cell count monitoring shows great potential for rapid susceptibility testing.

## Introduction

Antibiotic resistance has become a global threat to human health [1]. ‘Super-bugs’ like XDR tuberculosis and NDM1 metallo-betalactamases have emerged and spread globally [2,3]. Infections caused by multidrug resistant microorganims are often difficult to treat or can not be treated at all with antibiotics considered safe enough for the patient. In sharp contrast to the aforementioned, the development of new antibiotics has ceased, as demonstrated by the approval of only two new antibacterial agents based on new molecular entities since 1998 (linezolid in 2000 and daptomycin in 2003) [4]. In light of these developments it seems clear that only very stringent use of antibiotics, for instance by antibiotic stewardship programmes, may delay the rise of resistance and ascertain time to stimulate research into new drugs in the years to come. Switching from empirical broad spectrum antibiotic therapy to targeted therapy as soon as possible is one of the cornerstones of antibiotic stewardship but depends on the rapid availability of antimicrobial susceptibility data. Currently, several automated antimicrobial susceptibility testing methods are available, of which the VITEK2® (bioMérieux, Marcy-l'Étoile, France) (VITEK) and the Phoenix® (BD Biosciences, Frankklin Lakes, NJ, USA) system provide the most rapid results (mean time-to-result 9 h) [5]. Translated into daily clinical practice, however, it means that switching from broad spectrum empirical therapy to small spectrum targeted therapy will only be instituted the following working day. It is reasonable to assume that an earlier switch to targeted antibiotic therapy will have a decreasing effect on the rise of antimicrobial resistance. More importantly, rapid susceptibility results will shorten the use of inappropriate antibiotics, leading to increased patient survival [6] and decreased costs [7]. In this article we evaluated the concept of bacterial cell count monitoring using flow cytometry as a tool to determine antimicrobial susceptibility in antibiotic broth-dilution series. In flow cytometry, microscopic particles are analysed by suspending them in a stream of fluid on which a single wavelength laser beam is focussed. Particles passing the laser beam scatter the light, which is detected by forward and sideward photosensitive detectors. Furthermore, fluorescence caused by dyes staining DNA, RNA or specific proteins can be detected using fluorescence detectors. The pattern of scattered light is predictive for the size and shape of the particle. Combined with fluorescence data, cells can be differentiated from other particles and studied for transformations over time. Currently, several analysers are capable of analysing substantial quantities of fluid in a very short amount of time. The Sysmex UF-1000*i* flow cytometer (UF) we used in our study was designed to perform a complete urine sediment analysis (including the presence of red and white blood cells, epithelial cells, bacteria, mucus, crystals and casts) in only 90 s. Although the principle of flow cytometry has already been applied to antimicrobial susceptibility testing since 1982 [8], all papers published so far studied light scattering patterns, cell-elongation by DNA/RNA content or differences in dead and viable cells. None of these studies considered the possibility of bacterial cell counting over time to detect an increase, equilibrium or decrease in the number of particles (bacteria). This process obviously precedes the development of turbidity caused by bacterial growth, on which present-day conventional methods such as the VITEK and Phoenix® systems are based. In this article we demonstrate the proof-of-principle of cell count monitoring for antimicrobial susceptibility testing, resulting in a significant decrease in time-to-result.

## Materials and Methods

Strains were selected to represent bacterial families of which the members are most frequently isolated in clinical samples: *Escherichia coli* (*E. coli*) representing the Enterobacteriaceae, *Pseudomonas aeruginosa* (*P. aeruginosa*) representing the non-fermenting bacteria, and *Staphylococcus aureus* (*S. aureus*) representing the *Staphylococci*. Amoxicillin (or piperacillin in the case of *P. aeruginosa*) and gentamicin were the chosen antibiotics to be evaluated because they represent frequently used empirical antibiotics in patients and have a completely different mode of action (cell-wall production interference vs. protein production interference). From each aforementioned species four strains, derived from clinical samples and identified both by VITEK2® (bioMérieux) and Biflex® MALDI-TOF mass spectrometer (Bruker Daltonics, Bremen, Germany), were included. Initial susceptibility testing was performed by VITEK using CLSI breakpoints. For each species one strain showing a minimal inhibitory concentration (MIC) for amoxicillin (or piperacillin) close to the susceptible/resistant breakpoint, one strain showing a MIC above the resistant breakpoint of amoxicillin (or piperacillin), one strain showing a MIC for gentamicin close to the susceptible/resistant breakpoint MIC and one strain showing a MIC above the resistant breakpoint for gentamicin was selected. Besides the VITEK analysis, susceptibility testing was also performed by E-test® (Etest) (bioMérieux), and by broth-macrodilution (BMD). VITEK and Etest susceptibility testing was performed according to the manufacturers’ guidelines, BMD was performed according to CLSI criteria [9]. CLSI criteria were chosen over EUCAST criteria because our laboratory was still using CLSI criteria at the time the study was performed. For each method of susceptibility testing, time from initial incubation to test result (time-to-result) was recorded.

Each strain was plated on a blood agar medium and incubated at 35°C ambient atmosphere for 18 h. The next day four colonies were suspended in 6 mL Muller Hinton (MH) broth and analysed by the Sysmex UF-1000*i* flow cytometer (UF). If necessary, colonies were added until the UF showed a bacterial count of at least 10 000 bacteria/μL but <50 000 bacteria/μL. These bacterial cell counts were chosen deliberately to ensure the experiment started with a bacterial concentration between about 50 and 200 bacteria/μL, as explained in the next section. The 6 mL suspension was diluted ten-fold by transferring 2222 μL to a flask containing 20 mL of MH broth. Subsequently, 1111 μL of this dilution was suspended in 11 flasks each containing 30 mL MH broth. Ten of these flasks contained the desired antibiotic in concentrations of 0.5, 1, 2, 4, 8, 16, 32, 64, 128 and 256 mg/L (after adding the suspension). The eleventh flask did not contain the antibiotic and served as a positive control (PC). A twelfth flask, without the antibiotic or the bacterial suspension, was added to serve as a negative control (NC). Each of the 12 flasks was divided over six aliquots, resulting in six (time-) series of 12 aliquots containing a positive control, a negative control and an antibiotic dilution series. All aforementioned steps were performed after vortexing thoroughly. One series (*t*0) was processed immediately by the UF to acquire a baseline bacterial cell count and to eliminate series with unacceptable starting distributions between the 12 aliquots. All other series were placed in an incubator at 35°C ambient atmosphere. Series *t*60, *t*90, *t*120, *t*180 and *t*240 were processed by the UF after 60, 90, 120, 180 and 240 min of incubation, respectively. Bacterial counts by the UF were plotted in Excel® (Microsoft, Redmond, WA, USA) bar-graphs. The results were compared with VITEK, Etest and BMD results. Discrepancies were determined according to FDA guidelines [10].

## Results

**FIG. 1:**
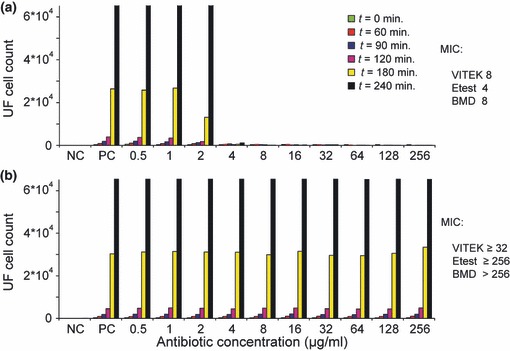
*Escherichia coli* strains exposed to amoxicillin. Bacterial cell counts per microlitre by flow cytometry over time for an amoxicillin-susceptible (a) and resistant (b) strain of *E. coli*. MIC, minimal inhibitory concentration; NC, negative control; PC, positive control; BMD, broth-macrodilution; Min, minutes of incubation at 35°C ambient atmosphere.

**FIG. 2:**
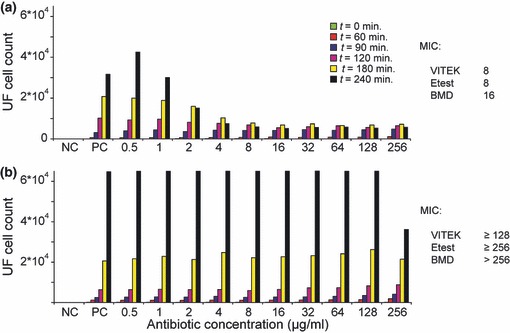
*Pseudomonas aeruginosa* strains exposed to piperacillin. Bacterial cell counts per microlitre by flow cytometry over time for a piperacillin-susceptible (a) and resistant (b) strain of *P. aeruginosa*. MIC, minimal inhibitory concentration; NC, negative control; PC, positive control; BMD, broth-macrodilution; Min, minutes of incubation at 35°C ambient atmosphere.

**FIG. 3:**
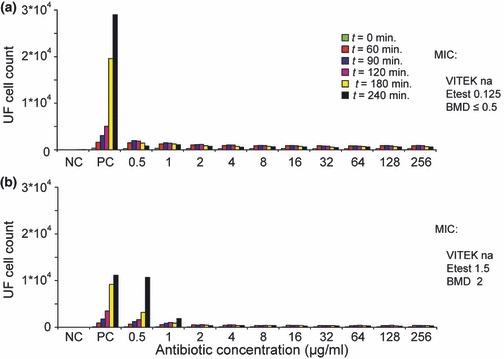
*Staphylococcus aureus* strains exposed to amoxicillin. Bacterial cell counts per microlitre by flow cytometry over time for an amoxicillin-susceptible (a) and resistant (b) strain of *S. aureus*. MIC, minimal inhibitory concentration; NC, negative control; PC, positive control; BMD, broth-macrodilution; Min, minutes of incubation at 35°C ambient atmosphere; na, not available.

Data obtained for *E. coli* and *S. aureus* strains exposed to amoxicillin and *P. aeruginosa* strains exposed to piperacillin are shown in Figures 1–3. The figures for exposure to gentamicin showed comparable results (data not shown). All three gold standard methods (VITEK, Etest and BMD) are commonly accepted as reliable procedures to determine or predict susceptibility. In our study, however, they were not uniformly in agreement with each other (Table 1). Theoretically, the MIC for a certain antibiotic should be the lowest antibiotic concentration at which no increase in cell count could be observed. Because antibiotics do not work instantly we anticipated that some bacterial growth would occur before the effect of the antibiotic could be detected. Therefore, we considered the MH-broth with the lowest antibiotic concentration showing a cell count reduction of at least 80% as compared with the positive control after 240 min as the predicted MIC by flow cytometry. This definition resulted in a 100% correct prediction of the MIC for all strains and all antibiotics according to the reference methods.To investigate whether susceptibility could be predicted at an earlier time-point, smaller reductions in cell count (i.e. 20%, 40% or 60%) were considered after shorter incubation periods. It appeared that a 100% correct prediction of the MIC could be made for *E. coli* already after 90 min of incubation if cell count was reduced by 60% as compared with the positive control. Also, in the case of *S. aureus* a 100% correct prediction could be made if cell count stayed 60% behind, albeit that this required 120 min of incubation. Even for *P. aeruginosa* the MIC could be 100% correctly predicted after 120 min if cell count was reduced by 40%. This lower percentage of ‘growth inhibition’ is understandable, as it is in line with the commonly known slower growth rate of *P. aeruginosa*. All MIC data obtained with the aforementioned cut-off values are shown in Table 1. According to FDA criteria, no minor, major or very major discrepancies were observed when flow cytometry was compared with each individual reference standard [10]. It has to be mentioned, though, that for the amoxicillin-susceptible *S. aureus* strain Etest showed an MIC of 0.125 whereas flow cytometry showed a decrease of >60% at an amoxicillin concentration of 0.5 mg/L. As we did not use lower amoxicillin concentrations in our study, this MIC found by Etest could not be confirmed by flow cytometry. Comparison of time-to-result data between the fastest currently accepted method (VITEK) and bacterial cell count monitoring are shown in Table 2.

**TABLE 1:** Comparison of MICs found by different methods

Strain	Figure no.	Antibiotic tested	VITEK MIC	Etest MIC	BMD MIC	Flow cytometry MIC
*E. coli*^a^	1a	Amoxicillin	8	4	8	4
*E. coli*^a^	1b	Amoxicillin	≥32	≥256	>256	>256
*E. coli*^a^	na	Gentamicin	≤1	0.5	≤0.5	1
*E. coli*^a^	na	Gentamicin	≥16	32	128	64
*P. aeruginosa*^b^	2a	Piperacillin	8	8	16	16
*P. aeruginosa*^b^	2b	Piperacillin	≥128	≥256	>256	>256
*P. aeruginosa*^b^	na	Gentamicin	≤1	1.5	2	1
*P. aeruginosa*^b^	na	Gentamicin	≥16	≥256	>256	>256
*S. aureus*^c^	3a	Amoxicillin	na	0.125	≤0.5	≤0.5
*S. aureus*^c^	3b	Amoxicillin	na	1.5	2	1
*S. aureus*^c^	na	Gentamicin	≤0.5	0.5	≤0.5	≤0.5
*S. aureus*^c^	na	Gentamicin	≥16	128	128	128

a Lowest antibiotic concentration showing 60% reduction in cell count as compared with the positive control after 90 min of incubation.

b Lowest antibiotic concentration showing 40% reduction in cell count as compared with the positive control after 120 min of incubation.

c Lowest antibiotic concentration showing 60% reduction in cell count as compared with the positive control after 120 min of incubation.

na, not available; BMD, broth-macrodilution.

**TABLE 2:** Comparison of time-to-result between VITEK and bacterial cell count monitoring using the optimal cut-off: for *E. coli* and *S. aureus*, lowest antibiotic concentration showing 60% reduction in cell count as compared with the positive control; cut-off for *P. aeruginosa*, lowest antibiotic concentration showing 40% reduction in cell count as compared with the positive control

Strain	Figure no.	Antibiotic tested (S/R)	VITEK (h:min)	Cell count monitoring (h:min)	Reduction in time-to-result (%)
*E. coli*	1a	Amoxicillin (S)	7:50	1:30	−80
*E. coli*	1b	Amoxicillin (R)	5:50	1:30	−74
*E. coli*	na	Gentamicin (S)	9:50	1:30	−85
*E. coli*	na	Gentamicin (R)	6:00	1:30	−75
*P. aeruginosa*	2a	Piperacillin (S)	13:50	2:00	−86
*P. aeruginosa*	2b	Piperacillin (R)	13:25	2:00	−85
*P. aeruginosa*	na	Gentamicin (S)	12:00	2:00	−83
*P. aeruginosa*	na	Gentamicin (R)	12:00	2:00	−83
*S. aureus*	3a	Amoxicillin (S)	na	2:00	na
*S. aureus*	3b	Amoxicillin (R)	na	2:00	na
*S. aureus*	na	Gentamicin (S)	8:25	2:00	−76
*S. aureus*	na	Gentamicin (R)	8:25	2:00	−76

na, antibiotic not avaliable on VITEK2 card; S, susceptible according to CLSI criteria; R, resistant according to CLSI criteria.

## Discussion

Our results clearly demonstrate the proof of principle of bacterial cell count monitoring for antimicrobial susceptibility testing. Antibiotic effects on susceptible strains are crystal clear after 240 min of incubation. This effect is definitely absent in the case of a resistant strain. Compared with the mean time-to-result of 9 h by currently accepted methods [5], cell count monitoring using the UF reduced the time-to-result by 55%. Surprisingly, analysis of the results showed that an effect of antibiotics on the increase of bacterial cell count could already be presumed after 60 min of incubation. Reduction in cell count proved to be capable of a 100% reliable forecast upon the actual MIC and was able to reduce the time-to-result to 90 min (in the case of *E. coli*) or 120 min (in the case of *S. aureus* and *P. aeruginosa*). These time-to-results will reduce the currently accepted standard by at least 74–86%. Although this tremendous reduction in time-to-result is appreciable the real advantage will be that the chance of prescribing appropriate targeted antibiotic therapy 1 day earlier increases significantly. This will not only reduce mortality [6] and costs [7], but may also decelerate the rise of resistance. The study we performed is not the first using flow cytometry to determine antimicrobial susceptibility. Nearly 40 papers have been published on this topic since the first publication in 1982 by Steen *et al.* [8]. During the 1990s flow cytometry was less developed than nowadays and comprised time consuming procedures using inefficient dyes on expensive analysers. Investigators studied the ratio of viable/dead cells, cell-elongation, fluorescence intensity, DNA/RNA content and cell morphology when selected bacteria were exposed to antibiotics. Like our study, most of these studies were able to demonstrate antibiotic effects on bacterial cells already within 1–2 h after exposure to antibiotics [11]. However, the procedures used were too impractical to be accepted in daily clinical practice. As a result, from 2000 on the interest in flow cytometry for this purpose waned, according to the number of publications in the international literature. None of these studies concluded that a change in bacterial cell count over time could be used as a more easy to perform and reliable predictor of antimicrobial susceptibility. In 1996 Walberg *et al.* [12] published a study including a graph (graph 4) showing that the broth containing an *E. coli* strain increased rapidly in bacterial count over time when the strain was incubated without ampicillin or with a sub-MIC concentration of ampicillin. When the same strain was incubated with antibiotic concentrations above the MIC no increase in bacterial count was observed at all. This difference was already visible after 40 min and became increasingly visible until the end of the experiment at 100 min. Although this remarkable finding was discussed in the results section, the authors did not mention it in the discussion section. Mason *et al.* [13] also reported an increase of bacterial cell count for the control and sub-MIC antibiotic concentration broths, whereas above-MIC concentrations cell counts remained stable. This effect, which was present at least after 60 min, was not clearly visible in their graph using a log particle counts/mL scale. Again, these authors did not refer to this finding in their discussion section. Nevertheless, these papers support our idea that bacterial cell count monitoring can be used as a very fast procedure to reliably predict antimicrobial susceptibility. The application of cell count monitoring may even go further as Cohen *et al.* [14] showed in 1989 that flow cytometry could reliably predict the presence of antimicrobial susceptibility for amikacin directly in clinical samples containing polyflora. They showed that amikacin susceptibility was reliably detected within 1 h in 92% of 13 clinical samples. All the aforementioned studies used protocols hampered by laborious procedures, suboptimal dyes and high costs. For this reason, more straightforward and easier to automate turbidity and colorimetric measuring techniques, used for instance in VITEK, gained the upper hand and became the standard procedure in daily laboratory practice. Nowadays, molecular-based detection of resistance genes or gene-complexes is becoming more and more popular, supposedly obviating the need for phenotypic susceptibility testing. These techniques are as yet time consuming and costly. Moreover, they need specialized technicians and equipment. The most important drawback, however, is the inability to detect new and unknown resistance mechanisms. Thus, molecular techniques may reliably demonstrate resistance, but cannot reliably predict susceptibility. In clinical practice, however, the latter is eventually decisive for the most appropriate antibiotic to be prescribed. In our opinion, phenotypic testing will therefore maintain its important role in antimicrobial susceptibility testing in the future. During the past decennia, flow cytometry techniques have improved considerably. The flow cytometer we used is designed to do a complete urine sediment analysis in relatively ‘contaminated’ solutions harbouring multiple kinds of bacteria, cells and debris instead of a bacterial count analysis in an otherwise clean fluid. Clearly, this analyser is over equipped and not specifically designed for our goal. Also, the exact polymethine fluorescent dye used for staining nucleic acids and proteins is patented and thus not known to us. We believe there is room for improvement, enabling even shorter time-to-results, when a less complicated and tailor-made flow cytometer is used. Besides flow cytometry, other techniques such as electrical micro-impedance spectroscopy [15] are being developed, which might even be better, quicker and cheaper to answer our central question: increase or not in bacterial cell count.

In conclusion, our study showed that the principle of bacterial cell count monitoring can reliably predict antimicrobial susceptibility in 90–120 min, decreasing the currently accepted time-to-result by 74–86% for *E. coli*, *S. aureus* and *P. aeruginosa* strains. This reduction of time could have a significant impact on mortality, costs and the rise of microbial resistance.
